# Estimating diabetes prevalence among NYC youth: Evidence from the Automated Student Health Record

**DOI:** 10.1371/journal.pone.0344345

**Published:** 2026-11-11

**Authors:** Sophia E. Day, Gabrielle Sorresso, Maria Fitzpatrick, Kevin Konty, Sophia White, Cristina Yunzal-Butler, Kira Argenio

**Affiliations:** 1 NYC Department of Health and Mental Hygiene, NYC Office of School Health, New York, New York, United States of America; 2 Jeb E. Brooks School of Public Policy, Cornell University, Ithaca, New York, United States of America; 3 Department of Economics, Jeb E. Brooks School of Public Policy, Cornell University, Ithaca, New York, United States of America; 4 Weill Cornell School of Medicine, New York, New York, United States of America; City College of New York, UNITED STATES OF AMERICA

## Abstract

We provide the first estimates of childhood type 1 and type 2 diabetes prevalence following the onset of the COVID-19 pandemic. Our analysis documents change in prevalence from 2010−11–2021−22, with a particular focus on trends from 2016−17 onward, using Automated Student Health Record (ASHR) data maintained by New York City Department of Health and Mental Hygiene (NYC DOHMH). We study non-homeschooled students aged 5−17 in grades pre-K to 12 enrolled in New York City Public Schools (NYCPS), resulting in an analytic sample of 2,167,033 unique students. Our main outcome measure is type 1 or 2 diabetes prevalence per 1,000 students each school year. In 2021−22, type 1 diabetes prevalence was highest among White students (2.73 per 1,000; 95% CI: 2.44, 2.97) and those aged 15−17 (2.24 per 1,000; 95% CI: 2.03, 2.45), with no significant increase since 2016−17 (*p* > 0.050). Meanwhile, we find type 2 diabetes increased nearly 40% from 2016−17–2021−22 (*p* < 0.050), with the highest prevalence among students with obesity (2.35 per 1,000; 95% CI: 1.96, 2.74). While type 2 diabetes has been rising for most demographic groups since 2016−17, we estimate that increases in type 2 diabetes prevalence are largest for older students, students of color, those with obesity, or those living in high-poverty areas, although most increases are not significantly different from other groups. Finally, we demonstrate that systems like ASHR can be a valuable resource for tracking chronic conditions in youth nationwide, underscoring its potential for broader public health monitoring.

## Introduction

Together, type 1 diabetes and type 2 diabetes are the third most common chronic childhood illness [1,2]. Though distinct in their causes and management, both illnesses raise the risk of current and future health complications for children [1,2]. Data on childhood diabetes prevalence are limited, primarily coming from the SEARCH for Diabetes in Youth study (SEARCH) [3]. These data show that while both type 1 and type 2 diabetes have risen since the early 2000s, type 2 diabetes has increased more rapidly [3]. While SEARCH has led the way in providing youth diabetes prevalence estimates, their estimates are only released every eight years (2001, 2009, and 2017) [3]. Post-pandemic studies on diabetes incidence —the rate of new diagnoses— mostly using small populations in medical settings, have also flourished as a way to understand diabetes in children over time [4–8]. However, these settings cannot account for changes in the populations visiting these centers post-pandemic onset, potentially biasing these estimates.

Given the importance of this topic and the increasing demand for frequent, subnational health data, we utilize Automated Student Health Record (ASHR) data to provide the first estimates of childhood type 1 and type 2 diabetes prevalence since the onset of the pandemic [9]. Described in detail below, ASHR is housed in a larger data system, the Student Population Health Registry (SPHR), which includes other demographic information about students and is maintained by the Office of School Health (OSH), a joint program of the New York City Department of Health and Mental Hygiene (NYC DOHMH) and New York City Public Schools (NYCPS) [10,11]. ASHR is used to document routine school-based services (e.g., student vision screenings, nurse visits), and has been used for syndromic surveillance of short-term acute disease outbreaks [12–15]. We pilot the use of ASHR for estimating chronic disease prevalence among NYCPS students. ASHR offers three key advantages over other common data sources used to track chronic diseases in children. First, it allows more frequent prevalence estimation than large-scale surveys and multi-center studies (e.g., SEARCH) [3]. Second, ASHR can be linked with rich sociodemographic and anthropometric details in SPHR, like BMI, neighborhood poverty, and home language, often not available in larger studies [16,17]. Finally, these data can pinpoint schools with high diabetes prevalence rates enabling efficient and timely resource deployment.

This study has three objectives. First, we estimate type 1 and type 2 diabetes prevalence among New York City public school students from 2016−17–2021−22, providing the first population-level estimates post-pandemic onset. Second, we identify which demographic subgroups have the highest diabetes prevalence to inform targeted prevention and management efforts. Third, we demonstrate the utility of ASHR data for chronic disease surveillance in children. By establishing ASHR’s capacity to track diabetes prevalence with greater frequency and geographic specificity than existing national studies, this work provides a model for timely, actionable public health monitoring that can guide resource allocation and intervention planning at the school and neighborhood level.

## Research design and methods

### Study population

Our diabetes diagnosis data come from ASHR, a live, school-based historical record system maintained by OSH at the NYC DOHMH, which our team analyzed between August 3, 2024 and January 17, 2025 [10,11]. ASHR is part of the SPHR data system, which includes student-level demographic and anthropometric information. Within ASHR, we restrict our sample to those enrolled in general or special education, and in grades pre-kindergarten (pre-K) through 12 from school years 2010−11–2021−22 (N = 12,393,572). We further limit to those aged 5−17 (N = 11,057,244), for whom we have a sufficient sample at each age. We exclude homeschooled children (N = 71,422) due to limited health information for this population. The analytic sample includes 2,167,033 individual students across all twelve years and 1,458,003 individual students from 2016−17 onward. This study was reviewed by the Cornell University IRB (Protocol IRB0147040) and deemed not-research.

The NYC DOHMH Institutional Review Board determined this study to be public health surveillance and therefore “deemed to not be research” (45 CFR 46.102.l.2) and exempt from the requirement for obtaining written informed consent.

### Variable description

ASHR tracks diabetes diagnoses in children through three reporting mechanisms. First, families submit the required Child and Adolescent Health Examination Form (CH-205) upon enrollment in NYCPS. This form is completed by a licensed healthcare provider and documents the student’s medical evaluation. An OSH clinician reviews the form to confirm the diagnosis. Second, families of children with diabetes may submit a Diabetes Medication Administration Form (DMAF), which provides the school nurse with instructions on diabetes management. All DMAFs are reviewed by an OSH clinician upon receipt for quality assurance. Clarifications or modifications to this document require confirmation with the prescribing provider. While students with a diabetes diagnosis are required to submit a DMAF annually, non-insulin-dependent students with type 2 diabetes and older students managing their own insulin may not submit one.

Third, students may have a diabetes diagnosis recorded in ASHR if the school is otherwise informed of a diagnosis by a healthcare provider [18]. All diabetes diagnoses in ASHR are reviewed by an OSH clinician, which helps ensure their accuracy. Once a student’s diabetes diagnosis is entered into ASHR, it remains for subsequent school years unless the student’s parent/guardian or provider notifies the school that the diagnosis is no longer accurate.

In ASHR, there are three small populations with non-standard diabetes diagnosis entries. First, 240 students switch between type 1 and type 2 diabetes across school years. Second, 180 students were, at least once, diagnosed with both type 1 and type 2 diabetes in the same year. In both scenarios, we assign students their most frequent diagnosis. When frequency is tied, we use the most recent single diagnosis as a tiebreaker.

While some students diagnosed with both type 1 and type 2 diabetes or who switch between the two diagnoses over time may represent rare diagnoses not recorded in ASHR, such as maturity-onset diabetes of the young, most cases are likely errors in diagnosis or data entry. After these adjustments, we observe 103 students with a type 2 diabetes diagnosis under age 10, though having this diagnosis before 10 is uncommon [19,20]. Most (62%) of these diagnoses result from assigning the modal diagnosis in the situations described above. We do not report type 2 diabetes prevalence for those under 10, as type 2 diabetes is rarely diagnosed before age 10 as it typically presents during puberty when physiologic insulin resistance peaks [21].

Student date of birth, sex, race/ethnicity, place of birth, language spoken at home, and home address are collected by the NYC DOE in SPHR through parent report. We classify students’ sex as male or female and race/ethnicity into six categories: Hispanic, non-Hispanic White (White), non-Hispanic Black (Black), Asian/Pacific Islander (Asian), Native American, and other (including multiple races). We categorize students’ place of birth as U.S.-born or foreign-born and classify home language into three categories: English, Spanish, or other.

We also use geocoded student addresses to determine the socioeconomic status of their residential census tract. Consistent with DOHMH guidelines, we classify tracts using the percentage of households below the federal poverty threshold (FPT) with 2008–2012 ACS data. Tracts are classified as low-poverty (<10% below FPT), medium-poverty (10%- < 20% below FPT), high-poverty (20%- < 30% below FPT), and very-high-poverty (≥30% below FPT) [22].

Additionally, in SPHR height and weight are recorded annually during NYC FITNESSGRAM assessments for students in K-12th grade. These measurements allow us to calculate age- and sex-specific body mass index (BMI) percentiles [16,17]. We classify students’ BMI into the following five categories using BMI percentile and 2022 Centers for Disease Control and Prevention growth charts: underweight (<5%), healthy weight (5%- < 85%), overweight (85%- < 95%), obesity (95%- < 120% of the 95th percentile), and severe obesity (≥120% of the 95th percentile) [23].

In a typical year, 13% of students lack complete height and weight data; however, due to the onset of the pandemic, data were more incomplete in 2019−20 compared to a typical year (46% missing height and weight data) and unavailable in 2020−21. Missing BMI values are imputed using data from the prior two years, reducing missing observations to 5.6% on average across all years. Fifteen percent of the 5.6% of missing observations are from students in special education schools, for whom participation in BMI measurement may not be required.

### Statistical analyses

All analyses were conducted using Stata version 16. Prevalence is estimated as the proportion of enrolled students with a diabetes diagnosis in each school year, multiplied by 1,000, and reported with 95% confidence intervals computed using the normal approximation to the binomial. Changes in prevalence between the 2016−17 and 2021−22 school years are estimated via ordinary least squares regression of the binary diabetes indicator on a year indicator, where the coefficient on the year indicator equals the difference in prevalence across the two years. Because the two samples we are comparing may contain children observed at both points in time, when conducting inference on changes over time, we cluster the standard errors at the student level. Given our large sample sizes, this has limited effect on inference.

To evaluate whether slight shifts in the demographic composition of NYCPS between 2016−17 and 2021−22 affect the estimated prevalence change, we present supplemental analyses using inverse probability weighting (IPW). We estimate the probability of being observed in the 2021−22 school year (versus 2016−17) using logistic regression with the following covariates: sex, age category, race/ethnicity, BMI category, residential poverty category, home language, and place of birth. Weights for 2016−17 observations are set to the inverse of the predicted probability of being in 2021−22, reweighting the 2016−17 population to match the 2021−22 demographic distribution. Weights for 2021−22 observations are set to one. The weighted analysis results are presented in the supplemental tables. Finally, we also compare our results to SEARCH’s national estimates [3].

## Results

Fig 1 shows the prevalence of type 1 and type 2 diabetes over time. Type 1 diabetes prevalence remains stable around 1.65 per 1,000 children. There does appear to be a small uptick post-pandemic onset; however, the prevalence between 2010−11 and 2021−22 is not clinical meaningful or significantly different (*p* > 0.050). In contrast, type 2 diabetes prevalence increased by 0.14 per 1,000 students (a 42% relative increase) from 2016−17–2021−22, and by 0.17 per 1,000 (59%) since 2010−11 (p < 0.050 for both)

**Fig 1:**
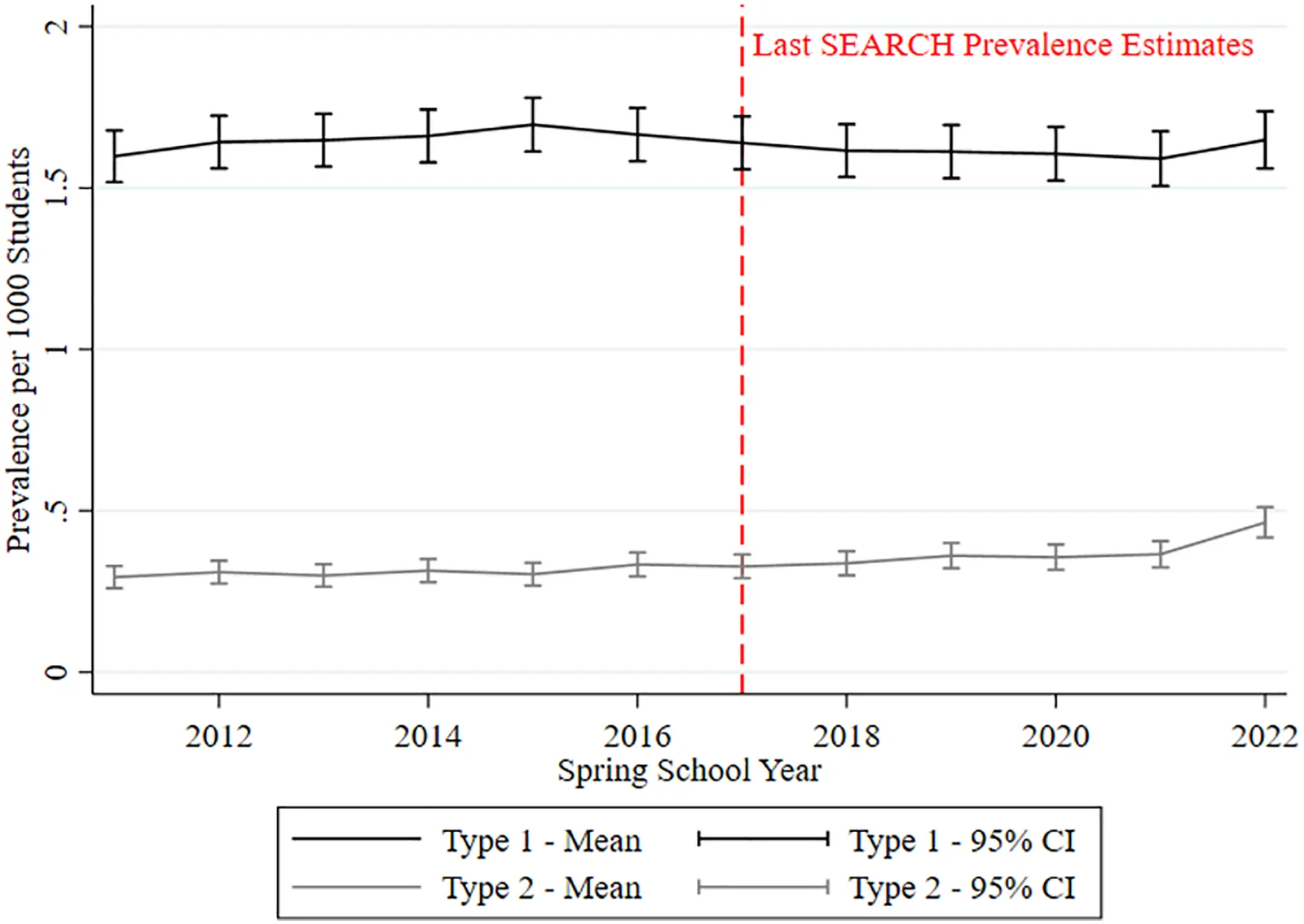
Diabetes prevalence over time, NYC Public School Students, ASHR data, 2016−17–2021−22 school years.

### Type 1 diabetes

Among 810,191 students in the 2021−22 school year, 1,336 students had type 1 diabetes, resulting in a prevalence of 1.65 per 1,000 (95% CI, 1.56, 1.74) (Table 1). This represents an absolute change of 0.01 per 1,000 students from 2016−17, when prevalence was 1.64 per 1,000 (95% CI, 1.56, 1.72), a difference that is not clinically or statistically meaningful (*p* > 0.050). Changes across all demographic subgroups were similarly small in absolute magnitude, with no group showing a clinically meaningful change in type 1 diabetes prevalence between the 2016−17 and 2021−22 school years (*p* > 0.050 for all groups but one).

**Table 1:** Type 1 Diabetes prevalence among New York City Public School Students in 2016−17 and 2021−22 and difference over time using ASHR/SPHR Data.

Group		2016−17 School Year		2021−22 School Year		2016−17 and 2021−22 Difference	P-Value
		Prevalence (CI)	N	Prevalence (CI)	N		
Total		1.64 (1.56, 1.72)	933,647	1.65 (1.56, 1.74)	810,191	0.01 (−0.09, 0.11)	0.85
Sex	Male	1.61 (1.50, 1.73)	480,147	1.74 (1.61, 1.87)	416,918	0.13 (−0.01, 0.27)	0.07
	Female	1.67 (1.55, 1.79)	453,500	1.55 (1.43, 1.68)	393,273	−0.12 (−0.25, 0.02)	0.10
Age	5-9	0.93 (0.84, 1.03)	369,562	0.96 (0.85, 1.08)	293,745	0.03 (−0.12, 0.18)	0.69
	10-14	1.93 (1.79, 2.08)	350,103	1.91 (1.76, 2.06)	316,093	−0.02 (−0.23, 0.19)	0.83
	15-17	2.38 (2.17, 2.59)	213,982	2.24 (2.03, 2.45)	200,353	−0.14 (−0.43, 0.15)	0.36
Race	Asian	0.74 (0.61, 0.87)	165,138	0.68 (0.55, 0.81)	154,318	−0.06 (−0.21, 0.09)	0.44
	Black	1.79 (1.61, 1.96)	218,450	1.83 (1.62, 2.04)	163,940	0.04 (−0.18, 0.27)	0.70
	Hispanic	1.60 (1.47, 1.73)	382,267	1.58 (1.44, 1.71)	338,348	−0.02 (−0.17, 0.12)	0.76
	Native Amer.	1.61 (0.77, 2.45)	8,697	2.47 (1.48, 3.46)	9,717	0.86 (−0.02, 1.74)	0.06
	White	2.59 (2.33, 2.85)	149,820	2.73 (2.45, 3.01)	129,637	0.14 (−0.17, 0.45)	0.38
	Other	0.54 (0.07, 1.01)	9,275	1.34 (0.74, 1.94)	14,231	0.80 (0.11, 1.48)	0.02
BMI^a^	Underweight	0.92 (0.63, 1.21)	41,233	0.96 (0.63, 1.29)	33,398	0.04 (−0.39, 0.46)	0.87
	Healthy Weight	1.77 (1.66, 1.89)	531,599	1.86 (1.73, 2.00)	411,562	0.09 (−0.06, 0.24)	0.23
	Overweight	1.73 (1.53, 1.94)	158,093	1.81 (1.58, 2.04)	130,332	0.08 (−0.22, 0.37)	0.61
	Obesity	1.47 (1.26, 1.69)	121,393	1.37 (1.15, 1.58)	109,129	−0.11 (−0.39, 0.17)	0.45
	Severe Obesity	1.15 (0.86, 1.43)	54,113	1.13 (0.86, 1.41)	59,084	−0.01 (−0.38, 0.36)	0.95
Residential Poverty^b^	0% - < 10%	1.86 (1.67, 2.05)	195,604	1.89 (1.69, 2.09)	178,868	0.03 (−0.20, 0.27)	0.78
	10% - < 20%	1.64 (1.49, 1.80)	257,455	1.58 (1.42, 1.75)	224,564	−0.06 (−0.25, 0.13)	0.52
	20% - < 30%	1.54 (1.37, 1.70)	222,648	1.54 (1.36, 1.72)	184,374	0.00 (−0.20, 0.21)	0.97
	30% − 100%	1.58 (1.42, 1.73)	253,073	1.70 (1.51, 1.88)	199,997	0.12 (−0.08, 0.32)	0.25
Place of Birth^c^	US	1.77 (1.68, 1.86)	776,155	1.78 (1.68, 1.88)	669,688	0.01 (−0.10, 0.12)	0.84
	Non-US	0.98 (0.83, 1.14)	156,916	1.02 (0.85, 1.19)	138,047	0.04 (−0.15, 0.23)	0.68
Home Language^d^	English	2.03 (1.91, 2.15)	533,415	2.03 (1.91, 2.16)	465,428	0.01 (−0.14, 0.15)	0.91
	Spanish	1.09 (0.95, 1.23)	220,610	1.04 (0.89, 1.18)	189,530	−0.05 (−0.21, 0.11)	0.54
	Other	1.17 (1.01, 1.33)	179,526	1.24 (1.06, 1.41)	154,948	0.07 (−0.12, 0.26)	0.47

^a^These are age and sex specific Body Mass Index categories. In 2016−17 school year: 27,216 observations missing; In 2021−22 school year: 66,686 observations missing

^b^In the 2016−17 school year: 4,867 observations missing; In the 2021−22 school year: 22,388 observations missing

^c^In the 2016−17 school year: 576 observations missing; In the 2021−22 school year: 2,456 observations missing

^d^In the 2016−17 school year: 96 observations missing; In the 2021−22 school year: 285 observations missing

In the 2021–22 school year, type 1 diabetes prevalence was higher for males (1.74 per 1,000; 95% CI, 1.61, 1.87) than females (1.55 per 1,000; 95% CI, 1.43, 1.68), an absolute difference of 0.10 students per 1,000, (*p* < 0.050). Prevalence also increased with age (*p* < 0.050): the prevalence during 2021–22 was 0.96 per 1,000 for those aged 5–9 (95% CI, 0.85, 1.08), 1.91 per 1,000 for those aged 10–14 (95% CI, 1.76, 2.06), and 2.24 per 1,000 for those aged 15–17 (95% CI, 2.03, 2.45), representing a 2.3-fold difference in prevalence between the youngest and oldest age groups. White students had the highest prevalence of any racial/ethnic group (2.73 per 1,000; 95% CI, 2.45, 3.01) (*p* < 0.050), followed by Native American students, who had a prevalence of (2.47 per 1,000; 95% CI, 1.48, 3.46), though the difference between these two groups is not clinically or statistically meaningful (*p* > 0.050).

In the 2021−22 school year, type 1 diabetes was least prevalent among underweight children (0.96 per 1,000; 95% CI, 0.63, 1.29). Compared to the healthy weight group (1.86 per 1,000), this represents an absolute difference of 0.90 per 1,000, which was lower than all other BMI categories (*p* < 0.050), except for those with severe obesity. Type 1 diabetes prevalence was higher in low-poverty neighborhoods (1.89 per 1,000; 95% CI, 1.69, 2.09) than in medium or high-poverty neighborhoods, with absolute differences of approximately 0.31 and 0.35 per 1,000 respectively, (p < 0.050). Prevalence was also elevated in very-high-poverty neighborhoods (1.70 per 1,000; 95% CI, 1.51, 1.88), though the absolute difference from other poverty levels was small and not statistically significant (p > 0.050). Additionally, prevalence was higher for U.S.-born children compared to foreign-born children, an absolute difference of 0.76 per 1,000, (*p* < 0.050) and for students who speak English at home versus students who speak Spanish or another language at home (*p* < 0.050).

### Type 2 diabetes

In the 2021−22 school year, 376 students had type 2 diabetes among the population of 810,191 children, resulting in a prevalence of 0.46 per 1,000 (95% CI, 0.42, 0.51) (Table 2). This represents an absolute increase of 0.14 per 1,000 students (a 42% relative increase) from a prevalence of 0.33 per 1,000 in 2016−17 (*p* < 0.050), corresponding to approximately 110 additional students with type 2 diabetes across NYCPS. In the 2021−22 school year, type 2 diabetes was more prevalent among females (0.52 per 1,000; 95% CI, 0.45, 0.59) than males (0.41 per 1,000; 95% CI, 0.35, 0.47), an absolute difference of 0.11 per 1,000 (*p* < 0.050). Since 2016−17, prevalence rates have risen by 0.17 per 1,000 for males (95% CI, 0.09, 0.23; a 71% relative increase) and 0.10 for females (95% CI, 0.02, 0.19; a 24% relative increase). In addition, both are statistically significant increases (*p* < 0.050).

**Table 2:** Type 2 Diabetes Prevalence among New York City Public School Students in 2016−17 and 2021−22 and Difference Over Time using ASHR/SPHR Data.

Group		2016−17 School Year		2021−22 School Year		2016−17 and 2021−22 Difference	P-Value
Mean & CI	N	Mean & C1	N
Total	Total	0.33 (0.29, 0.36)	933,647	0.46 (0.42, 0.51)	810,191	0.14 (0.08, 0.19)	<0.01
Sex	Male	0.24 (0.20, 0.29)	480,147	0.41 (0.35, 0.47)	416,918	0.17 (0.09, 0.23)	<0.01
	Female	0.42 (0.36, 0.48)	453,500	0.52 (0.45, 0.59)	393,273	0.10 (0.02, 0.19)	0.01
Age	5-9	–	–	–	–	–	–
	10-14	0.41 (0.34, 0.48)	350,103	0.54 (0.46, 0.62)	316,093	0.13 (0.03, 0.24)	0.01
	15-17	0.65 (0.55, 0.76)	213,982	0.97 (0.84, 1.11)	200,353	0.32 (0.14, 0.49)	<0.01
Race/Ethnicity	Asian	0.18 (0.11, 0.24)	165,138	0.21 (0.14, 0.28)	154,318	0.03 (−0.05, 0.12)	0.47
	Black	0.57 (0.47, 0.67)	218,450	0.88 (0.74, 1.03)	163,940	0.32 (0.16, 0.48)	<0.01
	Hispanic	0.33 (0.28, 0.39)	382,267	0.52 (0.44, 0.59)	338,348	0.18 (0.10, 0.27)	<0.01
	Native Amer.	0.23 (−0.09, 0.55)	8,697	0.72 (0.19, 1.25)	9,717	0.49 (−0.05, 1.03)	0.08
	White	0.15 (0.09, 0.21)	149,820	0.09 (0.04, 0.14)	129,637	−0.05 (−0.12, 0.02)	0.13
	Other	0.11 (−0.10, 0.32)	9,275	0.35 (0.04, 0.66)	14,231	0.24 (−0.13, 0.62)	0.20
BMI^a^	Underweight	0.05 (−0.02, 0.12)	41,233	0.09 (−0.01, 0.19)	33,398	0.04 (−0.08, 0.16)	0.51
	Healthy Weight	0.08 (0.06, 0.11)	531,599	0.11 (0.08, 0.14)	411,562	0.02 (−0.01, 0.06)	0.19
	Overweight	0.34 (0.25, 0.43)	158,093	0.53 (0.40, 0.65)	130,332	0.19 (0.04, 0.34)	0.01
	Obesity	0.54 (0.41, 0.67)	121,393	0.83 (0.66, 1.01)	109,129	0.29 (0.08, 0.50)	0.01
	Severe Obesity	2.18 (1.79, 2.57)	54,113	2.35 (1.96, 2.74)	59,084	0.17 (−0.34, 0.68)	0.51
Residential Poverty^b^	0% - < 10%	0.17 (0.12, 0.23)	195,604	0.28 (0.20, 0.36)	178,868	0.11 (0.01, 0.20)	0.02
	10% - < 20%	0.32 (0.25, 0.39)	257,455	0.35 (0.27, 0.42)	224,564	0.02 (−0.07, 0.12)	0.61
	20% - < 30%	0.39 (0.30, 0.47)	222,648	0.58 (0.47, 0.69)	184,374	0.19 (0.07, 0.31)	<0.01
	30% − 100%	0.40 (0.32, 0.48)	253,073	0.69 (0.57, 0.81)	199,997	0.29 (0.16, 0.42)	<0.01
Place of Birth^c^	US	0.35 (0.31, 0.39)	776,155	0.50 (0.44, 0.55)	669,688	0.15 (0.09, 0.21)	<0.01
	Non-US	0.22 (0.14, 0.29)	156,916	0.31 (0.22, 0.40)	138,047	0.09 (−0.01, 0.20)	0.07
Home Language^d^	English	0.37 (0.32, 0.42)	533,415	0.56 (0.49, 0.62)	465,428	0.18 (0.11, 0.26)	<0.01
	Spanish	0.34 (0.26, 0.41)	220,610	0.43 (0.34, 0.53)	189,530	0.10 (−0.01, 0.20)	0.07
	Other	0.18 (0.12, 0.25)	179,526	0.23 (0.15, 0.30)	154,948	0.04 (−0.04, 0.13)	0.34

^a^These are age and sex specific Body Mass Index categories. In 2016−17 school year: 27,216 observations missing; In 2021−22 school year: 66,686 observations missing

^b^In the 2016−17 school year: 4,867 observations missing; In the 2021−22 school year: 22,388 observations missing

^c^In the 2016−17 school year: 576 observations missing; In the 2021−22 school year: 2,456 observations missing

^d^In the 2016−17 school year: 96 observations missing; In the 2021−22 school year: 285 observations missing

In the 2021−22 school year, type 2 diabetes prevalence was higher among students aged 15−17 (0.97 per 1,000; 95% CI, 0.84, 1.11) compared to those aged 10−14 (0.54 per 1,000; 95% CI, 0.46, 0.62), a 1.8-fold difference in absolute prevalence. Prevalence increased from the 2016−17 school year for both age groups: by 0.13 per 1,000 children (95% CI, 0.03, 0.24; a 32% relative increase) for those aged 10−14 and by 0.32 per 1,000 children (95% CI, 0.14, 0.49; a 51% relative increase) for those aged 15−17. In 2021−22, prevalence was highest among Black children (0.88 per 1,000; 95% CI, 0.74, 1.03) compared to all other racial/ethnic groups (<0.050), except for Native American children (0.72 per 1,000; 95% CI, 0.19, 1.25), whose wide confidence interval precludes a precise comparison to Black children From the 2016−17–2021−22 school year, type 2 diabetes increased significantly for Black children (0.32 per 1,000 increase; 95% CI, 0.16, 0.48) and for Hispanic children (0.18 per 1,000 increase; 95% CI, 0.10, 0.27). The 0.32 per 1,000 increase among Black children and 0.18 per 1,000 increase among Hispanic children represent 56% and 55% relative increases respectively, the largest relative increases of any racial/ethnic group. Although the increase for Native American children was also large (0.49 per 1,000 increase; 95% CI, −0.05, 1.03), it is less precisely estimated (*p* > 0.050) due to the smaller sample size.

In 2021−22, type 2 diabetes was least prevalent for underweight and healthy weight children. The prevalence in these groups was 0.09 per 1,000 (95% CI, −0.01, 0.19) and 0.11 per 1,000 (95% CI, 0.08, 0.14), respectively, with no significant or clinically meaningful changes since 2016−17 (*p* > 0.050). Prevalence was higher for those with a higher BMI, 0.53 per 1,000 for students with overweight (95% CI, 0.40, 0.65), 0.83 per 1,000 for students with obesity (95% CI, 0.66, 1.01), and 2.35 per 1,000 for students with severe obesity (95% CI, 1.96, 2.48), representing a more than 21-fold difference between healthy weight and severely obese students. Between 2016−17 and 2021−22, prevalence increased for those with overweight (0.19 per 1,000 increase; 95% CI, 0.04, 0.34; a 56% relative increase) and obesity (0.29 per 1,000 increase; 95% CI, 0.08, 0.50; a 54% relative increase). While the estimated increase between 2016−17 and 2021−22 for students with severe obesity was relatively large (0.17 per 1,000 increase; 95% CI, −0.38, 0.73), it was not statistically different from zero due to the relatively small sample (*p* > 0.050).

Type 2 diabetes prevalence was higher for children in high and very-high-poverty neighborhoods compared to those in medium or low-poverty neighborhoods (*p* < 0.050), as well as for those born in the U.S. and those speaking English at home (*p* < 0.050). Increases in prevalence were observed across most demographic groups when looking by poverty; the groups showing no meaningful increase were those in medium-poverty neighborhoods, those born outside the U.S., and those speaking a non-English language at home (*p* > 0.050).

### SEARCH comparison

We compare prevalence from our initial year of available data (2010−11) with the SEARCH 2009 study, and our 2016−17 rates with SEARCH from 2017 (Table 3) [3]. In 2010−11, our results show a slightly higher, but comparable, prevalence of type 1 diabetes (1.60 per 1,000; 95% CI, 1.52, 1.68) to SEARCH in 2009 (1.48 per 1,000; 95% CI, 1.44, 1.52) (p < 0.050). We find no statistically significant change in type 1 diabetes prevalence between 2010−11 and 2016−17, while the SEARCH study shows a large increase in prevalence between 2009 and 2017, with prevalence growing to 2.15 per 1,000 in 2017 (95% CI, 2.10, 2.20). Our 2010−11 type 2 diabetes prevalence rates (0.29 per 1,000; 95% CI, 0.26, 0.33) are comparable to SEARCH in 2009 (0.34 per 1,000; 95% CI, 0.31, 0.37). Our type 2 diabetes prevalence does not significantly change from 2010−11–2016−17, while prevalence in SEARCH nearly doubles from 0.34 to 0.67 per 1,000 (95% CI, 0.63, 0.70) (p < 0.050). Despite these differences, our results generally align with SEARCH regarding which demographic groups have the highest diabetes prevalence rates.

**Table 3:** Comparison of diabetes prevalence in ASHR/SPHR Data (2010−11 and 2016−17) and SEARCH Study in 2009 and 2017.

		Diabetes Type 1				Diabetes Type 2			
Group		ASHR Data 2010−11	SEARCH Study 2009	ASHR Data 2016−17	SEARCH Study 2017	ASHR Data 2010−11	SEARCH Study 2009	ASHR Data 2016−17	SEARCH Study 2017
Total		1.60 (1.52, 1.68)	1.48 (1.44, 1.52)	1.59 (1.51, 1.67)	2.15 (2.10, 2.20)	0.29 (0.26, 0.33)	0.34 (0.31,0.37)	0.27 (0.24, 0.30)	0.67 (0.63, 0.70)
Gender	Male	1.58 (1.47, 1.70)	1.48 (1.43, 1.54)	1.57 (1.46, 1.68)	2.19 (2.13, 2.26)	0.22 (0.18, 0.26)	0.26 (0.23, 0.30)	0.19 (0.16, 0.23)	0.51 (0.47, 0.56)
	Female	1.61 (1.50, 1.73)	1.48 (1.42, 1.54)	1.61 (1.50, 1.72)	2.11 (2.04, 2.18)	0.37 (0.32, 0.43)	0.42 (0.38, 0.47)	0.35 (0.30, 0.40)	0.82 (0.77, 0.88)
Age	5-9	1.05 (0.95, 1.15)	1.17 (1.10, 1.25)	0.92 (0.83, 1.01)	1.33 (1.26, 1.41)	–	–	–	–
	10-14	1.90 (1.75, 2.04)	1.95 (1.86, 2.04)	1.91 (1.77, 2.05)	2.91 (2.80, 3.02)	0.33 (0.27, 0.39)	0.15 (0.13, 0.18)	0.37 (0.31, 0.43)	0.29 (0.26, 0.32)
	15-17/19^a^	2.01 (1.83, 2.19)	2.42 (2.32, 2.53)	2.28 (2.08, 2.48)	3.93 (3.81, 4.06)	0.57 (0.47, 0.67)	0.54 (0.49, 0.59)	0.58 (0.48, 0.68)	1.04 (0.98, 1.11)
Race	Asian	0.72 (0.58, 0.86)	0.50 (0.42, 0.61)	0.72 (0.59, 0.85)	0.76 (0.67, 0.85)	0.19 (0.12, 0.26)	0.35 (0.26, 0.48)	0.16 (0.10, 0.22)	0.59 (0.49, 0.72)
	Black	1.56 (1.41, 1.71)	1.29 (1.18, 1.41)	1.65 (1.50, 1.81)	2.18 (2.04, 2.33)	0.45 (0.37, 0.53)	0.95 (0.82, 1.10)	0.40 (0.33, 0.48)	1.80 (1.62, 2.00)
	Hispanic	1.65 (1.53, 1.78)	0.96 (0.89, 1.04)	1.59 (1.47, 1.71)	1.56 (1.48, 1.64)	0.28 (0.23, 0.33)	0.45 (0.39, 0.53)	0.29 (0.23, 0.34)	1.03 (0.94, 1.12)
	Native Amer.	1.16 (0.23, 2.09)	0.30 (0.22, 0.42)	1.48 (0.70, 2.25)	0.56 (0.43, 0.73)	0.00 (0.00, 0.00)	1.22 (0.98, 1.52)	0.21 (−0.08, 0.50)	1.63 (1.32, 2.03)
	White	2.48 (2.22, 2.74)	1.86 (1.80, 1.92)	2.50 (2.25, 2.75)	2.79 (2.72, 2.87)	0.15 (0.09, 0.21)	0.14 (0.12, 0.17)	0.12 (0.06, 0.17)	0.20 (0.17, 0.23)

Source for SEARCH Data: Lawrence JM, Divers J, Isom S, et al. Trends in Prevalence of Type 1 and Type 2 Diabetes in Children and Adolescents in the US, 2001–2017. *JAMA.* 2021;326 [8]:717–727. https://doi.org/10.1001/jama.2021.11165

^a^We report for those aged 15–17 while SEARCH reports prevalence for those aged 15–19.

## Conclusion

We present the first diabetes prevalence estimates in children post-pandemic onset and document changes over time. Our study highlights the utility of ASHR for monitoring and identifying trends in chronic disease prevalence. We find the prevalence of type 1 diabetes was stable between 2010−11 and 2021−22, contrasting with SEARCH, which found increases between 2009 and 2017, and other studies showing increased incidence of type 1 diabetes post-pandemic [3–8]. Similar to previous studies, we also find that type 2 diabetes prevalence increased after 2020, especially among older children, children of color, those with higher BMI, or living in high-poverty areas, although many of these increases are not significantly different from other groups. A portion of the post-pandemic increase in type 2 diabetes prevalence may be attributed to persistent lifestyle changes among youth driven by the pandemic, such as increased sedentary behavior [24,25]. However, assessing the reasons for the increase in type 2 diabetes we document is beyond the scope of this paper.

The 42% relative increase in type 2 diabetes prevalence corresponds to approximately 113 additional students with type 2 diabetes across NYCPS, concentrated disproportionately among older students, students of color, and those with higher BMI. These are populations already at elevated cardiometabolic risk, and even modest absolute increases in prevalence translate to meaningful demands on school-based care resources and portend greater long-term complication burden.

In 2010−11, our results were aligned with national SEARCH results from 2009 [3]. However, SEARCH finds large increases in both type 1 and type 2 diabetes in the following eight years, differing from our findings of no increase for type 1 diabetes and a smaller increase in type 2 diabetes. This could be due to different geographic samples as previous research has noted potential geographic differences in diabetes rates by region [26,27]. Additionally, while diabetes rates may have increased nationally, they may not have increased to the same extent among our sub-population, highlighting the need for both national and subnational studies.

Relatedly, we present ASHR as a valuable data source for real-time diabetes measurement. While national studies may have significant time lags and, as noted, may not be representative of trends in local areas, school-level data systems like ASHR can provide real-time, subnational tracking of chronic illnesses. These data can help practitioners spatially identify chronic disease clustering and efficiently deploy resources. Using ASHR, we can efficiently identify schools with a high percentage of students diagnosed with diabetes. This can be critical for timely direction of resources to the school, such as trainings to help staff recognize the signs of high and low blood sugar, or providing additional nurses to help facilitate student diabetes management plans [28].

While the population studied in ASHR is large, it is not representative of all children in NYC or the national population. Additionally, student demographics have changed slightly over time in NYCPS. To address this, we present results in the appendix using inverse probability weighting to ensure demographic balance across the 2016−17 and 2021−22 school years. This reweighting does not change our broad findings. However, our diabetes data rely on family reporting, so prevalence differences across demographic groups may in part reflect reporting gaps rather than true differences. Further, our post-pandemic onset findings may be biased downward due to reduced reporting during school closures. Lower form completion among Black and Hispanic families, for example, may bias diabetes prevalence downward, underestimating racial disparities. Nonetheless, our differences in prevalence rates across demographic groups align with SEARCH, which uses different data collection methods. Finally, we cannot be certain whether the uptick in type 2 diabetes prevalence reflects actual population changes or increased care-seeking and diagnosis due to heightened post-pandemic health awareness. However, this potential increase in awareness is not unique to our study and would also affect data from medical facilities. In sum, enhancing efforts to identify all children with diabetes would improve the utility of school datasets for tracking trends. Given the limits of ASHR, future research should expand on our work using diverse data sources with frequent reporting on large populations.
